# An atypical HLH transcriptional regulator plays a novel and important role in strawberry ripened receptacle

**DOI:** 10.1186/s12870-019-2092-4

**Published:** 2019-12-27

**Authors:** Laura Medina-Puche, Félix J. Martínez-Rivas, Francisco J. Molina-Hidalgo, José A. Mercado, Enriqueta Moyano, Antonio Rodríguez-Franco, José L. Caballero, Juan Muñoz-Blanco, Rosario Blanco-Portales

**Affiliations:** 1 Departamento de Bioquímica y Biología Molecular, Edificio Severo Ochoa C-6, Campus Universitario de Rabanales y Campus de Excelencia Internacional Agroalimentario CEIA3, Universidad de Córdoba, Córdoba, Spain; 2 Present Address: Shanghai Center for Plant Stress Biology (PSC), Shanghai Institutes of Biological Sciences, Chinese Academy of Sciences, Shanghai, China; 3 Present Address: VIB-UGent Center for Plant Systems Biology, Ghent, Belgium; 4 Instituto de Hortofruticultura Subtropical y Mediterránea La Mayora (IHSM-UMA-CSIC), Departamento de Biología Vegetal, Universidad de Málaga, Málaga, Spain

**Keywords:** Atypical HLH, Fruit ripening, PRE1, Strawberry

## Abstract

**Background:**

In soft fruits, the differential expression of many genes during development and ripening is responsible for changing their organoleptic properties. In strawberry fruit, although some genes involved in the metabolic regulation of the ripening process have been functionally characterized, some of the most studied genes correspond to transcription factors. High throughput transcriptomics analyses performed in strawberry red receptacle (*Fragaria x ananassa*) allowed us to identify a ripening-related gene that codes an atypical HLH (*FaPRE1*) with high sequence homology with the PACLOBUTRAZOL RESISTANCE (PRE) genes*.* PRE genes are atypical bHLH proteins characterized by the lack of a DNA-binding domain and whose function has been linked to the regulation of cell elongation processes.

**Results:**

*FaPRE1* sequence analysis indicates that this gene belongs to the subfamily of atypical bHLHs that also includes *ILI-1* from rice, *SlPRE2* from tomato and *AtPRE1* from *Arabidopsis*, which are involved in transcriptional regulatory processes as repressors, through the blockage by heterodimerization of bHLH transcription factors. *FaPRE1* presented a transcriptional model characteristic of a ripening-related gene with receptacle-specific expression, being repressed by auxins and activated by abscisic acid (ABA). However, its expression was not affected by gibberellic acid (GA_3_). On the other hand, the transitory silencing of *FaPRE1* transcription by agroinfiltration in receptacle produced the down-regulation of a group of genes related to the ripening process while inducing the transcription of genes involved in receptacle growth and development.

**Conclusions:**

In summary, this work presents for the first time experimental data that support an important novel function for the atypical HLH *FaPRE1* during the strawberry fruit ripening. We hypothesize that FaPRE1 modulates antagonistically the transcription of genes related to both receptacle growth and ripening. Thus, FaPRE1 would repress the expression of receptacle growth promoting genes in the ripened receptacle, while it would activate the expression of those genes related to the receptacle ripening process.

## Background

During the processes of development and ripening of soft fruits, many metabolic pathways that are responsible for organoleptic properties are differentially expressed. In strawberry fruits, it is very well established that the increase in the ABA/auxins ratio triggers the transcription of many ripening-related genes involving the different organoleptic properties such as aroma, color, taste and softening [1–10]. However, with the exception of some transcriptional factors (TFs), the functional characterization of genes involved in the regulation of these metabolic pathway is very scarce until now. Thus, it has been described the function played by a few transcription factors. *FaMYB10,* a R2R3 MYB TF related to the secondary metabolism, is described as a ripening-related master regulatory gene of the structural flavonoid/phenylpropanoid metabolic pathway genes [9]; and the *EMISSION OF BENZENOIDS II* (*FaEOBII*), a positive regulator of flavonoids/phenylpropanoids volatile-related genes, regulates the *CINNAMYL ALCOHOL DEHYDROGENASE* (*FaCAD1*) and the *EUGENOL SYNTHASE 2* (*FaEGS2*), which controls the production of eugenol, a volatile phenylpropanoid, in ripe strawberry receptacles [8]. Recently, a DOF-type TF (FaDOF2) has also been identified as a positive regulator of eugenol biosynthesis in ripened strawberry receptacle. Both FaEOBII and FaDOF2 seem to act synergistically in the activation of the *FaEGS2* gene transcription [11]. In this way, an ERF-MYB TF complex regulates furaneol biosynthesis by means of a quinone reductase transcription regulation [12]. The functional role played by FcMYB1, another R2R3 MYB TF, was also characterized. This TF acts as regulator of the branching point of the anthocyanins/proanthocyanidins biosynthesis [13]. Also, *FaGAMYB* has been described as a regulator in the transition from vegetative growth to ripening process [14]. In addition, it was demonstrated that the transient down-regulation of the C-type MADS-box TF expression *(SHATTERPROOF-like* gene*; FaSHP*) gave rise to a slightly shorter delay in the time required to reach the pink stage of ripening [7]. Besides, transcription of several ripening-related genes as well as the content of several metabolites was altered in these transiently modified fruits [7]. It was proposed that SCARECROW-LIKE 8 (FaSCL8) could modulate the transcription regulation of genes related to the flavonoid/anthocyanin biosynthesis, probably through their influence on *FaMYB10* gene expression [15]. Moreover, *FaMYB44.2* has been proposed to interact with *FaMYB10* in sucrose accumulation, which would have an impact on the ripening process [16]. On the other hand, four TFs (FaMYB9/FaMYB11, FabHLH3 and FaTTG1) have been described as positive activators of genes that are involved in the proanthocyanidins (PAs) biosynthesis in strawberry immature fruits [17]. A comparation between a mutant whited coloured strawberry and a red natural one has discovered some TF potentially involved in the anthocyanin biosynthesis [18].

High throughput transcriptomics analyses previously performed by our group [19] have allowed us to identify a ripening-related gene that codes an atypical HLH (*FaPRE1*) belonging to the basic helix-loop-helix/helix-loop-helix (bHLH/HLH) TFs family. *FaPRE1* was selected for its expression characteristics: a) ripening-related; b) receptacle-specific; c) negatively regulated by auxins, and d) induced by ABA [19]. According to their DNA-binding ability, these proteins are classified into two groups; DNA-binding bHLH (bHLH) and non-DNA-binding bHLH (HLH) proteins, also called atypical HLH [20–24]. bHLH TFs contain two clearly differentiated domains, a basic domain located at the amino terminus of the proteins, which contains 13–17 basic amino acids, and an HLH region, located at the carboxy terminus that comprises two amphipathic α-helices which are rich in hydrophobic amino acids and are connected by a loop of variable length. The basic domain gives the transcription factor the ability to bind to the DNA [24–26] while the presence of the HLH motif confers the ability to establish homo- or heterodimeric interactions with other bHLH proteins, which is essential for DNA recognition and DNA-binding specificity [22, 24]. On the contrary, HLH proteins are particularly diverged at the basic region, that usually lacked critical sequences for a proper DNA binding domain and, in consequence, they did not present DNA-binding ability [25]. HLH proteins may dimerize with other bHLH proteins [24, 27–29], thus acting as negative regulators of bHLH protein action through the formation of heterodimers. This interaction will avoid bHLH protein to interact with other bHLH and, in this way, with their corresponding *cis* sequences on the DNA [24, 29–34].

Several studies have shown that atypical HLH proteins play important regulatory roles in hormone signaling and cell elongation [20, 35–38], light signaling [39], vascular and fruit development [30, 34] or grain size [40, 41]. In this sense, functional analysis identified AtPRE1 (*Arabidopsis* PACLOBUTRAZOL RESISTANCE 1), an atypical HLH protein that plays an activator role of genes that respond to gibberellin, presumably downstream of DELLA proteins [20]. AtPRE1 also regulates organ elongation in response to BRs [31, 32]. Thus, AtPRE1 interacts with IBH1 (ILI1 binding bHLH 1), another atypical HLH that negatively regulates ACE1 (Activator of Cell Elongation 1). When AtPRE1 interacts with IBH1, it prevents its binding to ACE1 and restores the transcription ability of ACE to induce cell elongation [31, 32]. Thus, this triantagonistic bHLH system, which is generally used for these transcriptional regulators to perform its function, seems to be important in determining the final size of plant cells [31, 32].

Furthermore, in tomato, the *PRE*-like gene *SlStyle2.1* controls both the elongation and length of floral style, and has also been related to the evolution of self-pollination flowers in cultivated varieties [42]. In all these cases, the balance of triantagonistic bHLH proteins might be important to determine both the size of plant cells and the regulation of cell elongation, acting downstream of multiple external and endogenous signals [31, 32, 43].

Very little is known about the role of bHLH/HLH regulators in fruit ripening. In fruits, only an atypical HLH has been described in tomato (*SlPRE2*) that seems to participate in the development of the immature fruit but not in the stages of fruit ripening [33, 34]. This transcriptional factor is predominantly expressed in the fruit development and the silencing of its transcription diminished fruit size due to a thinning of the fruit pericarp [34]. Furthermore, *SlPRE2* transcription was GA_3_-inducible in immature green fruits. The authors suggest that SlPRE2 may regulate fruit size through the regulation of the cell expansion [34].

However, the specific role played by the bHLH/HLH in the fruit ripening process is not known. In this paper, we present the functional characterization, along the ripening of the strawberry receptacle, of an atypical HLH protein (FaPRE1). The transcription pattern of this gene is receptacle specific and clearly inducible along the ripening stages. In addition, the *FaPRE1* transcription is regulated positively for the internal concentration of abscisic acid (ABA) in the receptacle but not for the content of GA_3_. The transitory silencing of *FaPRE1* transcription by agroinfiltration in receptacle produced the down-regulation of a group of genes related to the ripening process while it induced the transcription of genes involved in receptacle growth. All these results indicate that *FaPRE1* plays a novel and important pivotal functional role along the receptacle ripening process differentially coordinating the antagonistic transcription of genes related to the receptacle growth and of those genes involved in receptacle ripening.

## Results

### *FaPRE* genes encode atypical HLH proteins

**Fig. 1:**
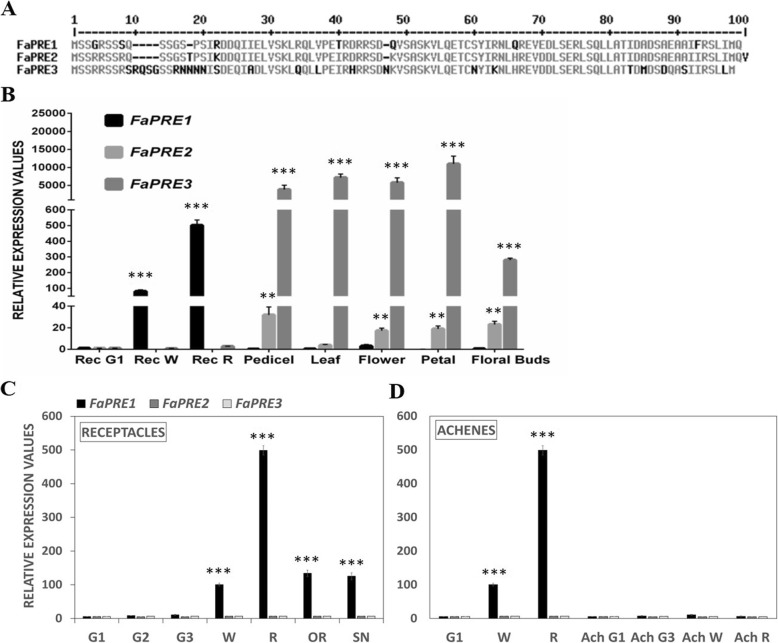
**a** Alignment of the predicted amino acid sequences of FaPRE1, FaPRE2 and FaPRE3 proteins. **b**-**d** Analysis by qRT-PCR of *FaPRE* genes expression in different tissues of *Fragaria* x *ananassa* “Camarosa” plants. **b** Analysis of *FaPRE* genes expression in some selected developing receptacles compared with vegetative tissues. Analysis of *FaPRE* genes expression in receptacles (**c**) and achenes (**d**) in different stages of development. Rec G1 and G1, small-sized green fruit; G2, middle-sized green fruits; G3, full-sized green fruit (G1 and G3: stages of development); Rec W and W, white stage; Rec R and R, ripe stage; OR, overripe stage; SN, senescent stage. Results were obtained using 3’UTR specific primers and quantification is based on Ct values. Relative expression values were calculated relative to receptacles G1 stage *C*t value, which was assigned an arbitrary value equal to unity. Values are mean ± SD of five independent experiments. Statistical significance with respect to the reference sample was determined by the Student’s *t*-test: **p < 0.01, ***p < 0.001

Bioinformatics analysis of the available Fragaria vesca (v2.0.a2) [44] and *Fragaria x ananassa* genome (v1.0-a1) [45] has allowed us to identify three *PRE* genes (*FaPRE*) in strawberry genome that we have named *FaPRE1* (*gene30478*), *FaPRE2* (*gene28320*) and *FaPRE3* (*gene03986*). The comparison of the deduced proteins from *FaPRE* genes had a 90% amino acid sequence identity among them (Fig. 1a). Phylogenetic analysis showed that FaPRE1, FaPRE2 and FaPRE3 proteins can be classified into the atypical HLH subgroup 16 of the 32 plant bHLH/HLH subfamilies [22] (Additional files 1, 2).

In addition, the analysis of the FaPREs amino acid deduced sequence with InterProScan software revealed that, as in other similar PRE-like proteins, FaPREs lack the basic region at the amino terminal end of the protein which is characteristic of the bHLH transcription factors and responsible for their specific DNA binding ability. The atypical HLHs interact with bHLHs transcription factors and, in this way, interfere with their regulatory activity by blocking its binding to the *cis*-regulatory sequences positioned on the gene promoters that regulate. In this sense, the existence of a putative helix-loop-helix (HLH) domain, which is important for the interaction with other HLH transcription factors, was observed in the three FaPREs deduced proteins (Additional file 3B). It is noteworthy that this HLH domain is highly conserved in all PRE family members from A. thaliana [20], as well as in other plants as rice and grape (Additional file 3C) [23, 46]. Using the Plant-mPLoc program (http://www.csbio.sjtu.edu.cn/cgi-bin/PlantmPLoc.cgi) to determine the bioinformatic prediction of FaPREs, a nuclear subcellular localization for these proteins was predicted (Additional file 3D), as has previously been described in other plant species [47, 48].

### FaPRE1 protein is located in nucleus

**Fig. 2:**
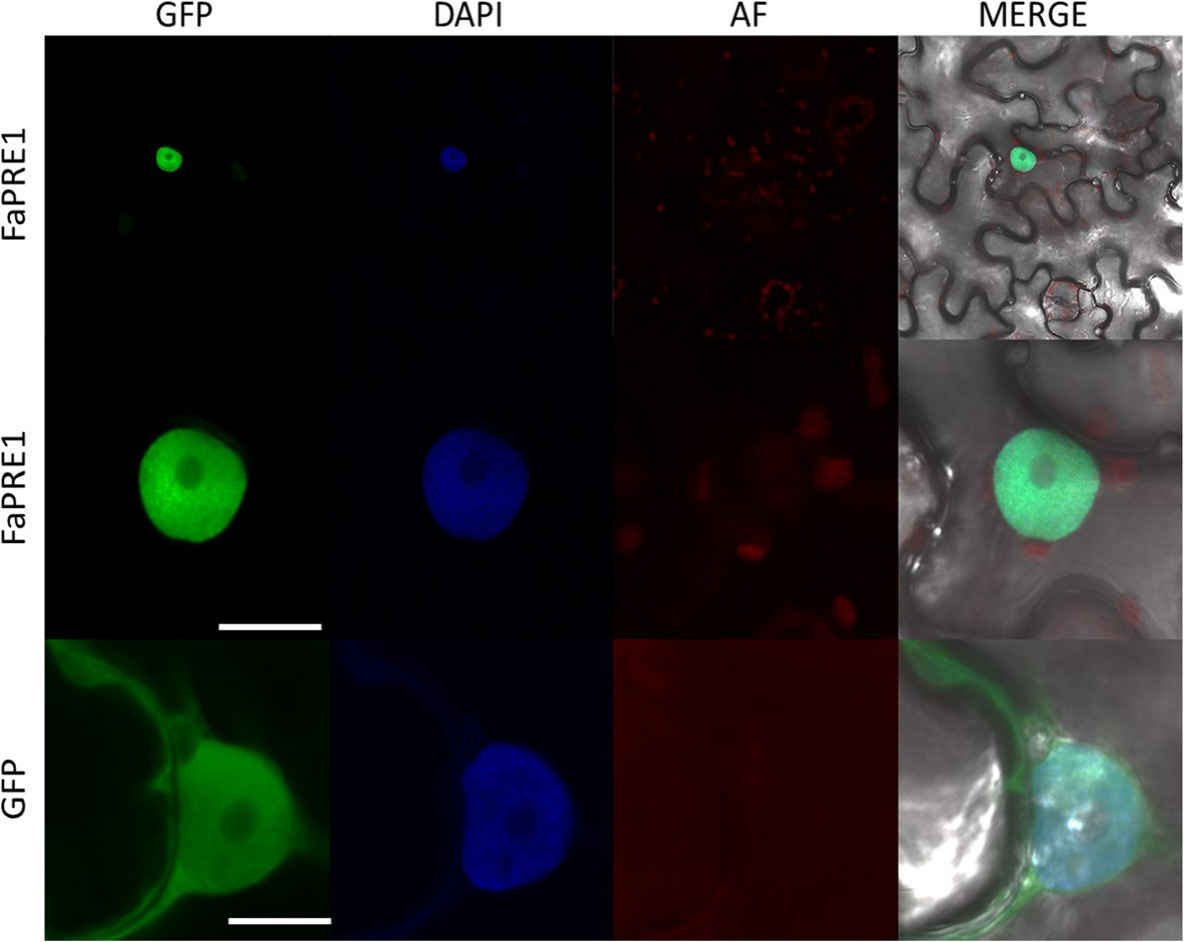
Subcellular localization of FaPRE1-GFP or free GFP upon transient expression in *Nicotiana benthamiana* leaves. Leaves from *N. benthamiana* were agroinfiltrated with translational constructs 35S-GFP-FaPRE1 and with 35S-GFP as control. Fluorescence signal detected using a confocal microscope. GFP, green fluorescent protein; DAPI, 4′,6-Diamidino-2-Phenylindole; AF, Autofluorescence; MERGE, merged view of the GFP and DAPI images. Scale bar: 5 μm

To confirm bioinformatics predictions related to the subcellular location of FaPRE1 protein, we carry out in vivo heterologous studies in *N. benthamiana*. For that, a N-terminal translational fusion protein construct between FaPRE1 and GFP proteins was driven under the control of a CaMV35S promoter. Confocal imaging analysis of the agroinfiltrated leaves indicated that the fusion protein co-localized with the nucleus marker DAPI (Fig. 2).

### The spatio-temporal expression of *FaPRE* genes indicates that *FaPRE1* is a ripening-related gene

qRT-PCR studies were performed to determine the spatial expression of the three *FaPRE* genes. Our analysis showed that *FaPRE2* and *FaPRE3* transcription was restricted to vegetative tissues with a scarce or negligible transcription in the receptacle. However, *FaPRE1* was almost exclusively expressed in ripe receptacle (Fig. 1b). For this reason, a more detailed spatio-temporal study of *FaPRE1* expression was carried out in the strawberry receptacle at different stages of growth and ripening. The data indicated that the amount of *FaPRE1* transcript increased steadily along the development and receptacle ripening stages, reaching their highest levels of transcription in the fully ripe stage (R). Afterwards, a slight decrease of transcript was observed in the overripe stage (OR), that was more pronounced in the senescent stage (SN), where only a low transcription level was detected (Fig. 1c). In contrast, transcript levels in achenes, corresponding to the different development and ripening stages, were negligible with respect to the values observed in the receptacle (Fig. 1d). Besides, the *FaPRE1* expression was not significant in vegetative tissues. All these data taken together suggest the participation of *FaPRE1* in the strawberry receptacle ripening process while *FaPRE2* and *FaPRE3* would develop their function in the vegetative tissues of the plant.

### Hormonal regulation of *FaPRE* genes transcription

**Fig. 3:**
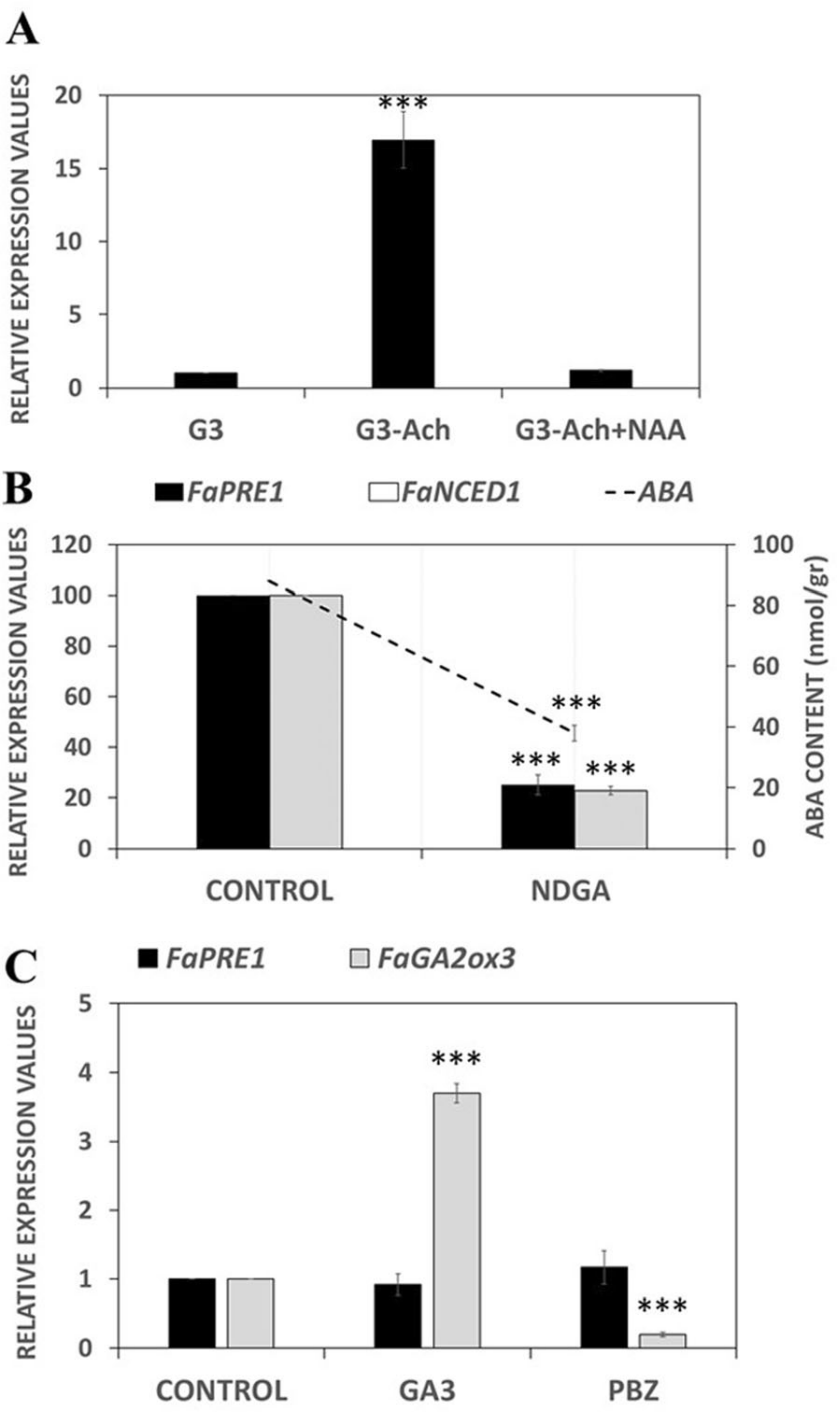
Hormonal effects in *FaPRE1* gene expression. **a** Analysis by qRT-PCR of the effects of removing achenes from G3 developing fruits on *FaPRE1* gene expression. **b** Analysis of *FaPRE1* and *FaNCED1* gene expression (bars) in G-W fruits treated with NDGA in both experimental situations; line indicates the ABA content in the analyzed fruits. **c** Analysis of the effects of gibberellins on *FaPRE1* and *FaGA2ox3* expression which was used as a control. The increase in the mRNA value was relative to the CONTROL *C*t value of each experiment. Values are mean ± SD of five independent experiments. Statistical significance with respect to the reference sample was determined by the Student’s *t*-test: ***p < 0.001

Considering that *FaPRE1* is a ripening-related gene, its regulation by auxins and ABA was studied. It has been previously reported that achenes removal from the surface of immature G3-stage fruits decreases the inner concentration of auxins in the receptacle, which induces the transcription of many ripening-related genes [9, 19]. Similarly, the *FaPRE1* transcription increased in de-achened receptacles (G3-achenes) with respect to that observed in control receptacles (G3) (Fig. 3a). As expected, this induction was abolished by the external application of the synthetic auxin IAA (Fig. 3a). Both results suggest that *FaPRE1* gene transcription was negatively regulated by the internal content of auxins in immature receptacles. On the other hand, and supporting the previous data, the transcription of *FaPRE1* decreased in receptacles where ABA production was diminished either by the inhibition of FaNCED1 enzymatic activity through the fruit treatment with NDGA or by the transitory silencing of the *FaNCED1* transcription (Fig. 3b) [9]. This differential hormonal expression pattern shows that, as in the case of many ripening-related genes, *FaPRE1* gene transcription is regulated, directly or indirectly, by the ratio ABA/auxins [19].

**Fig. 4:**
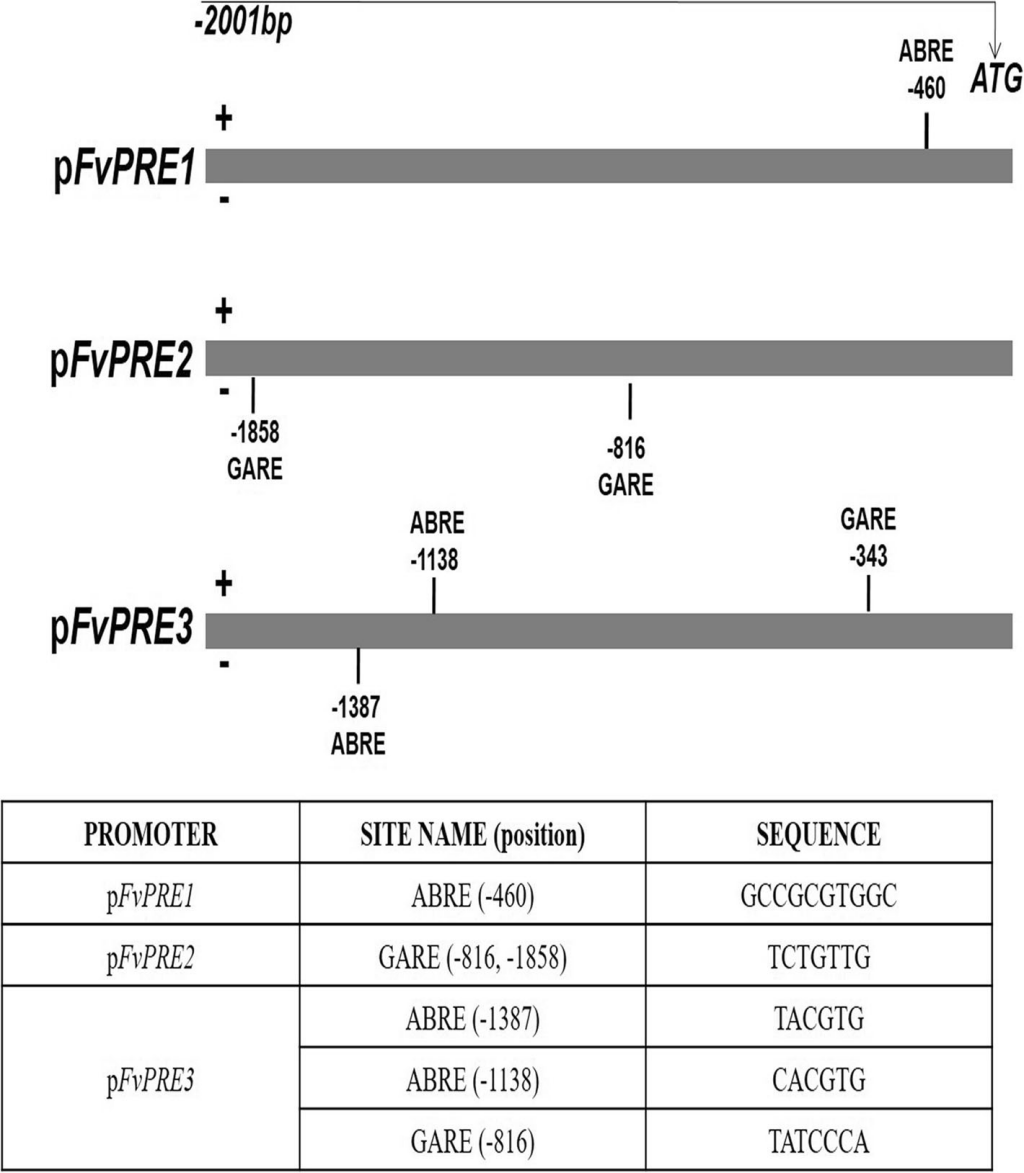
Schematic diagram of *FvPRE1*, *FvPRE2* and *FvPRE3* promoters from *Fragaria vesca.* The bar on top indicates the length of the promoter fragment relative to the ATG codon. ABRE and GARE motif are marked within their position in promoter sequence

In A. thaliana*,* it has been previously demonstrated that gibberellic acid (GA) regulates cell elongation through the increase of *AtPRE1* transcription [20]. On the other hand, *SlPRE2* also shows an important role in the cell enlargement along tomato fruit development through GAs [34]. In strawberry fruit receptacles, although endogenous GA content has been measured along receptacle development and ripening [49, 50], the relationship between GAs and fruit ripening has not been established. To determine whether *FaPRE1* transcription is under the control of GAs, strawberry fruit were injected with gibberellic acid (GA_3_) or paclobutrazol (PBZ), a compound that blocks gibberellin biosynthesis. Interestingly, in both cases, no significant changes were detected in *FaPRE1* transcription between treated fruits versus untreated control fruits while *GA2ox3*, a control gene related with the GA degradation in strawberry fruit, was induced by GAs and repressed by PBZ treatment respectively (Fig. 3c). Besides, no phenotypic changes were observed in treated fruits compared to controls (data not shown). These results discard that gibberellins affect the *FaPRE1* gene transcription in ripe fruits. This fact was reinforced by the bioinformatic analysis of the p*FaPRE1* promoter that showed the absence of GARE *cis*-regulatory sequences (DNA recognition sites of gibberellin response) in this promoter (Fig. 4).

On the contrary, in vitro strawberry plants treated with GA_3_ showed morphological changes that resulted in elongated plants while PBZ treated-plants displayed a dwarfed phenotype (Additional file 4). The analysis of *FaPRE* genes transcription in leaves, pedicel and root of the treated versus untreated plants showed that *FaPRE2* increased its transcription in all the analyzed tissues from GA_3_ treated-plants while *FaPRE3* transcription did not vary with respect to the control (Additional file 4B-D). In addition, *FaPRE2* and *FaPRE3* transcription was significantly reduced in all tissues in the presence of PBZ (Additional file 4 B-D). This suggests that both *FaPRE2* and *FaPRE3* are under the regulation of GA_3_ and probably play an active role in gibberellin signaling in vegetative tissues but not in fruits. Moreover, the promoter analysis of both genes presents GARE-motifs. In the p*FaPRE2* promoter region two GARE-motifs were present, whereas in p*FaPRE3* only one was present (Fig. 4). These data not only support the idea that the *FaPRE2* and *FaPRE3* transcription is regulated by gibberellins but relate the transcript level of each gene in response to this hormone with the number of GARE-motifs identified in their promoter sequences. Furthermore, these results support the proposal that the transcription of *FaPRE1* is independent of GA_3_ levels.

### High-throughput transcriptional analysis of transgenic receptacle where the *FaPRE1* transcription was silenced

Considering that FaPRE1 is a transcriptional co-regulator, to determine the putative functional role that *FaPRE1* plays along the ripening process, we proceeded to transitorily silence its transcription in ripened fruit by RNAi-*FaPRE1* agroinfiltration approaches. RNAi-*FaPRE1* silenced receptacles did not shown any phenotypical changes when compared to control receptacles (data not shown). Using a custom-made oligo-based microarray platform [19], a transcriptomic comparison between transgenic receptacle, where the *FaPRE1* transcription was silenced, versus control receptacles was carried out (Additional files 5, 6) and the obtained data were validated by qRT-PCR (Additional file 7). The transcriptomic results and their comparison with red receptacle transcriptomes [19], showed that the transcription of 227 genes was down-regulated in *FaPRE1* silenced ripen receptacles, out of which160 (70%) were also ripening-related genes (Additional files 8, 9). By way of contrast, the transcription of 276 genes was up-regulated in RNAi-*FaPRE1* receptacles, out of whom 211 (76%) were overexpressed in immature strawberry receptacles (Additional files 8, 10).

Among the ripening related genes whose transcription was downregulated in RNAi*-FaPRE1* ripen receptacles, we found transcription factors as *FaMyb10* (*gene31413*) and *FaEOBII* (*gene28435*) [8, 9] (Additional files 9, 10). Both TFs, with a regulatory role in the flavonoid/phenylpropanoid pathway during ripening, were significantly down-regulated in transgenic receptacle with *FaPRE1* transcription silenced (Table 1). The same behavior was shown by other genes whose function is described in strawberry during its ripening process, such as *gene21638* (*FaPG1*, *polygalacturonase-1* [51]) and *gene31030* (*FaRGlyaseI*, *rhamnogalacturonate lyase-1* [52]), which synthesize hydrolytic enzymes related with the cell wall dismantling during the ripening; *gene28407* (*FaQR*, *Quinone oxidoreductase* [53]), *gene07931* (*FaAAT2*, *Alcohol acyl transferase-2* [6]) and *gene34009* (*FaAAT1*, *Alcohol acyl transferase-1* [54]), which synthesize enzymes involved in the biosynthesis of esters that contribute to the final aroma of the fruit; and *gene14611* (*FaF3H*, *Flavanone 3-hydroxylase* [55]), *gene20700* (*FaCAD1*, *cinnamyl alcohol dehydrogenase-1* [56]) and *gene25260* (*FaEGS2, Eugenol synthase-2* [57]), related to the phenylpropanoids biosynthesis in strawberry ripe fruit (Table 1). All these results seem to indicate that *FaPRE1* gene might have a regulatory function in the strawberry ripening process.

**Table 1:**
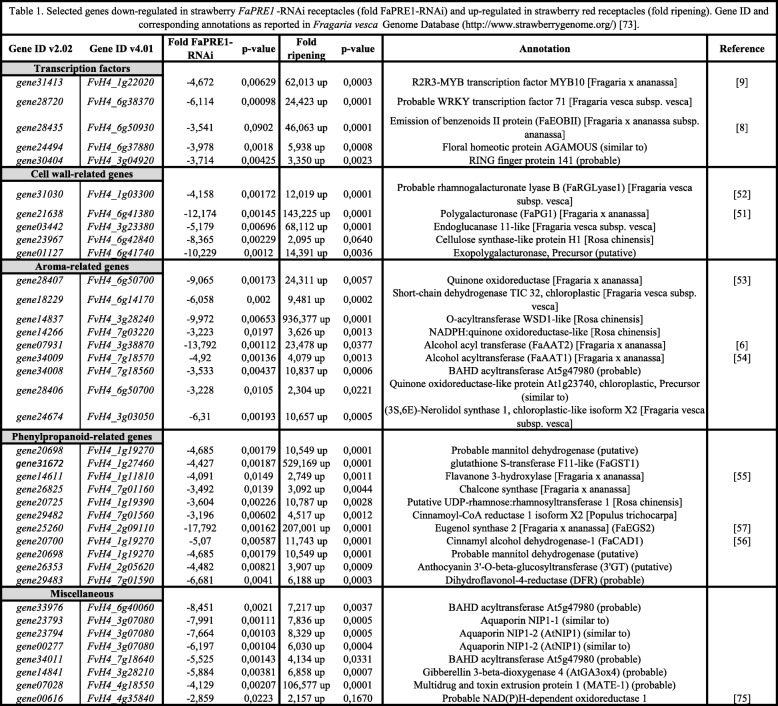
Selected genes down-regulated in strawberry FaPRE1-RNAi receptacles (fold FaPRE1-RNAi) and up-regulated in strawberry red receptacles (fold ripening). Gene ID and corresponding annotations as reported in *Fragaria vesca* Genome Database (https://www.rosaceae.org/) [73]

On the other hand, the *FaPRE1* silencing induced the expression of genes whose transcription was higher in immature green receptacles, in the development and growth stages, but not in ripening stages. Most of these genes are related with the metabolism and remodeling of the cell wall, both vital processes for the fruit growth and development. Thus, the *FaPRE1* silencing induced clearly the transcription of the *gene01986* and *gene02631* that encode a *Xyloglucan endotransglucosylase/hydrolase* and a *Xyloglucan glycosyltransferase* respectively (Table 2). These genes could be related with the hydroxylation and reconnection of xyloglucan fragments during the wall growth [58]. Similarly, *gene23429* (*Pectate lyase 12*) and *gene04435* (*Expansin-A1*) are also up-regulated in *FaPRE1*-RNAi receptacles. *PLs* and *Expansins* have been related to cell elongation and cell wall extensibility [58] (Table 2). In addition, the transcription of *gene20426* and *gene26607* was also induced in the same receptacles. Both genes encode beta-glucosidases, enzymes that are potentially involved in cellulose degradation [58] (Table 2). Otherwise, the transcription of *gene09384, gene11861* and *gene24005,* that encode an *Auxin efflux carrier*, an *Auxin-responsive protein IAA27*, and an *Indole-3-acetic acid-amido synthetase GH3.1* respectively, was additionally over-expressed in *FaPRE1-*RNAi receptacles (Table 2). These three genes are related to the response to auxins which is the hormone that regulates the strawberry receptacle growth and development [59].

**Table 2:**
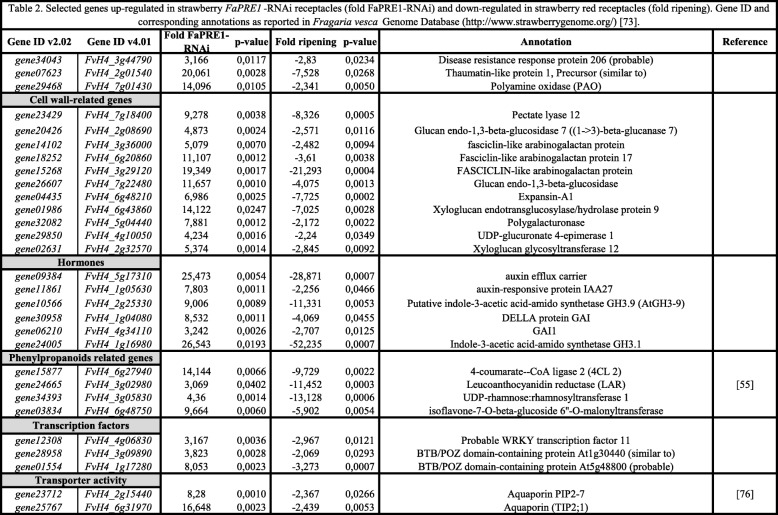
Selected genes up-regulated in strawberry FaPRE1-RNAi receptacles (fold FaPRE1-RNAi) and down-regulated in strawberry red receptacles (fold ripening). Gene ID and corresponding annotations as reported in *Fragaria vesca* Genome Database (https://www.rosaceae.org/) [73]

## Discussion

In this article, we present the functional characterization of the *FaPRE1*, a gene belonging to the strawberry *FaPRE* family (*FaPRE1*, *FaPRE2* and *FaPRE3*), which are the putative orthologous of the *AtPRE* genes from *Arabidopsis thaliana.*

*FaPRE1* gene was classified as member of the subfamily of atypical bHLHs by lacking a DNA binding domain. In this subfamily are also included both the *ILI-1* gene from rice [32], *SlPRE2* from tomato [33], as well as the members of the *Arabidopsis PRE* family [22] (Additional files 1, 2, 11). AtPRE1 [20], ATBS1 [39], PGL1 and APG [40, 60], and IBH1 [31] which are involved in transcriptional regulatory processes as repressors, through the blockage by heterodimerization of bHLH transcription factors. As expected by bioinformatic analysis, a nuclear localization of FaPRE1 (Fig. 2, Additional file 3D) supports their relationship with transcriptional regulatory processes. According to our experimental data the FaPRE1 could play a similar role in strawberry fruit ripening process.

### *FaPRE1* and fruit ripening

*FaPRE1* presented an expression model characteristic of a ripening-related gene, with transcription values negligible in both immature receptacles and vegetative tissues, but high in ripened stages. In strawberry, this is a common transcription pattern that is shared by the vast majority of ripening-related genes [19]. This model of expression is characterized by being a) ripening-related; b) receptacle-specific; c) negatively regulated by auxins, and d) induced by ABA. *FaPRE1* follows these criteria since the amount of *FaPRE1* transcript increases along receptacle ripening (Fig. 1c) and is preferentially expressed in mature red receptacle (Fig. 1b-c). Otherwise, the *FaPRE1* transcription was also negatively regulated by auxins but positively by ABA (Fig. 3a-b). The spatial-temporal and the hormonal transcription profile of *FaPRE1* are in agreement with the above-mentioned criteria and are also in accordance with the proposal of [59], who suggested that the ABA/auxins ratio determines the transition from the development to the ripening stage in the strawberry receptacle. Thus, auxins would be produced in immature achenes and released to the receptacle promoting its growth but preventing premature ripening. Afterwards, the auxin production would be arrested and subsequently the endogenous biosynthesis of ABA in the receptacle would be stimulated increasing the ABA/auxins ratio and thus promoting the ripening process [59]. This proposal was experimentally demonstrated recently [2, 5, 9]. A similar transcription pattern has been found in other ripening-related genes that encode transcription factors such as *FaMYB10* [9], *FaEOBII* [8] or *FaDOF2* [11]. Furthermore, it has been reported that *PRE*-like genes are under the positive regulation of GAs [20, 43]. That is not the case of the *FaPRE1* gene since the treatment of receptacles with GA_3_ did not result in an increase of its expression, unlike the*GA2ox3* control gene, discarding any involvement of this hormone in the regulation of *FaPRE*1 transcription in ripe strawberry fruit (Fig. 3c). This assumption is reinforced by the absence of regulatory sequences response to GAs (GARE-motifs) in the p*FvPRE1* promoter (Fig. 4). However, these motifs are present in the p*FvPRE2* and p*FvPRE3* promoters, two genes that have a specific expression of vegetative tissues. All these expression data suggest that *FaPRE1* plays a different physiological role than *FaPRE2* and *FaPRE3*, mainly focused on the process of fruit ripening.

In soft fruits, with the exception of tomato, the functional role played by *PRE*-like genes during the fruit ripening process has not been studied. Very recently, the relationship between a *PRE*-like atypical HLH gene (*SlPRE2*) and the growth of tomato fruit has been established [33, 34]. Thus, in tomato immature fruit, *SlPRE2* seems to have a repressor role of chlorophyll accumulation and chloroplast development. In addition, it represses the transcription of genes involved in the carotenoid’s biosynthesis during the fruit ripening process [33]. However, *SlPRE2* presents a different expression pattern from that observed for *FaPRE1*. Thus, while *SlPRE2* was expressed in both fruit and vegetative tissues as root, young leaf, mature leaf, senescent leaf, flower and sepal, the *FaPRE1* transcription was restricted to the ripened receptacle. In tomato vegetative tissues, the highest transcription levels were found in young leaf and in flowers where the elongation processes are more active. This is not the case of *FaPRE1*, whose transcription is mainly limited to the final stages of receptacle ripening, in which the processes of cellular elongation are not significant. Besides, *SlPRE2* was expressed strongly in small tomato immature fruit, but its transcript level decreased with the growth, although at a later point its transcription increased slightly along the ripening process [33, 34]. On the contrary, the *FaPRE1* transcription raised continuously throughout the receptacle ripening. The expression data suggest that FaPRE1 plays a physiological function different from that played by *SlPRE2* in tomato fruit. Otherwise, the *SlPRE2* overexpression (35S-*SlPRE2*) in tomato fruits gave rise to a decrease of both chlorophyll and carotenoid content in unripe and ripe fruits respectively. This fact was accompanied by a down-regulation of the transcript levels of genes related to chlorophyll metabolism and light signaling as GLK2, HY5, RbcS and Cab7 in fruits. Additionally, the transcription of genes involved in the biosynthesis of carotenoids such as phytoene synthase1 (PSY1), phytoene desaturase (PDS), and ζcarotene desaturase (ZDS) was significantly down-regulated in 35S-*SlPRE2* transgenic ripe fruits, with a concomitant reduction of lycopene content [33]. These findings indicated that *SlPRE2* regulates the chlorophyll and carotenoid content by repressing the expression of these chlorophyll and carotenoid biosynthetic genes. Besides, *SlPRE2* determine fruit size probably through a pathway GA_3_-dependent that regulate the pericarp cell expansion [34]. However, in the strawberry ripening process, the regulatory *FaPRE1* function seems to be quite different from that of *SlPRE2*. Certainly, the comparative analysis carried out between the *FaPRE1*-RNAi and control receptacles transcriptomes have shown that *FaPRE1* plays a dual function regulating the transcription of two groups of genes whose expression models are antagonistic. One of these groups includes those genes that are ripening-related and mainly expressed in the receptacle during the fruit ripening process, while the other group contains those genes that have an expression profile that is more related to the vegetative growth of the receptacle (Additional files 9, 10).

Among the genes whose transcription may be influenced by *FaPRE1* in ripened receptacles, we found genes involved in several metabolic processes related to the organoleptic properties of fruit. For instance, genes involved in the regulation of the transcription of those genes belonging to the flavonoid/phenylpropanoid metabolism and that codes two R2R3 MYB transcriptional factors as *FaMYB10* and *FaEOBII* [8, 9]. We have previously demonstrated that FaMYB10 regulates the transcription of most of the Early-regulated Biosynthesis Genes (EBGs) and Late-regulated Biosynthesis Genes (LBGs) involved in the flavonoid/phenylpropanoid pathway, including flavonol-3-hidroxylase (*FaF3H*)*, chalcone synthase* (*FaCHS*)*, dihidroflavonol reductase* (*DFR*), *cinnamoyl -CoA reductase* (*FaCCR*), *cinnamyl alcohol dehydrogenase* (*FaCAD1*), and *eugenol synthase-2* (*FaEGS2*) [9]. Also, FaMYB10 regulates the *FaEOBII* expression, which in turn regulates the transcription of the gene that encodes the FaEGS2, an enzyme involved in the biosynthesis of the phenylpropanoid volatile eugenol [8, 57]. The transcription of all these genes was also down-regulated in *FaPRE1*-RNAi receptacles (Table 3). These results suggest that FaPRE1 would play an important regulatory role of the phenylpropanoids pathway, probably through the regulation of the *FaMYB10* transcription through the sequestering of a bHLH whose function would be to suppress the gene expression of this FT in immature receptacle.

**Table 3:**
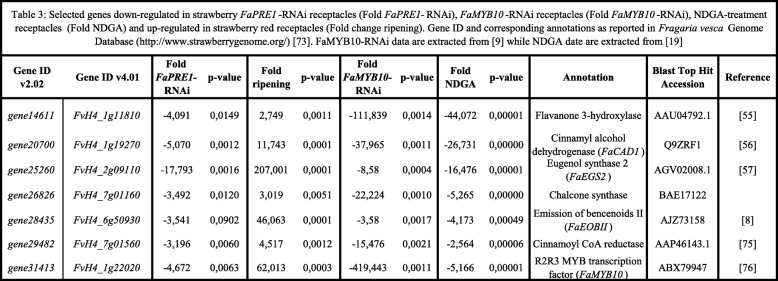
Selected genes down-regulated in strawberry *FaPRE1*-RNAi receptacles (Fold change FaPRE1-RNAi), *FaMYB10*-RNAi receptacles (Fold change *FaMYB10*-RNAi) and NDGA-treatment receptacles and up-regulated in strawberry red receptacle (Fold change ripening). Gene ID and corresponding annotations as reported in *Fragaria vesca* Genome Database (https://www.rosaceae.org/) [73]. *FaMYB10*-RNAi data are extracted from [9] while NDGA data are extracted from [19]

In addition, the expression of cell wall-related genes as *FaPG1* and *FaRGlyaseI* [51, 52], that has been previously demonstrated that are involved in the cell wall disassembly, was also down-regulated in RNAi-*FaPRE1* receptacles (Table 1).

The same behavior was shown by other genes whose function has been described in strawberry during its ripening process and related with aroma production. For instance, one of the genes whose transcription was down-regulated in RNAi-*FaPRE1* strawberry receptacles was the ripening-related *FaQR* gene, that encodes a quinone oxidoreductase*.* We have demonstrated that this enzyme is crucial for the furaneol (4-hydroxy-2,5-dimethyl-3(2H)-furanone; HDMF) biosynthesis, one of the most important components of the strawberry fruit aroma [61]*.* Besides, the transcription of genes which encode two enzymes involved in the biosynthesis of the key esters that contribute to the final aroma of the ripened fruit, such as FaAAT1 (*Alcohol acyl transferase-1*) and FaAAT2 (*Alcohol acyl transferase-2*) [6, 54], was also down-regulated in RNAi-*FaPRE1* strawberry receptacles. All these genes share a common expression profile as receptacle ripening-related genes (Table 1). In general, all these results seem to indicate that *FaPRE1* gene has a regulatory function in the strawberry ripening process.

As mentioned above, PREs are HLH proteins which lack the basic domain required for DNA binding but dimerize with DNA binding factors bHLH to inhibit their DNA binding ability [20, 33, 62, 63]. In this sense, we hypothesize that FaPRE1 might exert its transcriptional regulatory properties through the formation of an inactive FaPRE1 HLH: bHLHa heterodimeric complex that would withdraw the amount of bHLHa available to form a homo or heterodimeric transcriptionally active complex. We propose that this heterodimerization should inhibit the formation of a putative heterodimeric (bHLHa:bHLHb) transcriptional activator. This complex would up-regulate the transcription of non-ripening related genes that are expressed specifically in the immature receptacle. These genes are related to the growth and development stages and must be silenced in ripened receptacles. On the contrary, in ripened receptacles, FaPRE1 would inhibit the formation of another bHLH heterodimeric negative regulatory complex that determines the down regulation of ripening-related genes, but in non-ripened immature receptacles. This repressor would be constituted by a heterodimer of two DNA binding basic helix-loop-helices (bHLHa:bHLHc). Thus, the formation of a repressor complex in ripened receptacle would be inhibited by sequestering one of the monomer partners (bHLHa). In this way, the repression of ripening-related genes would be avoided thus facilitating its expression.

A similar but not identical mechanism of interaction between *FaPRE*-like genes and bHLHs in response to different signals, including light, temperature, BRs and GAs, has been described [31, 32, 43]. For instance, three *PRE* genes (PRE1, PRE3/ATBS1, PRE6/KIDARI), positively regulate organ elongation in response to GAs, BRs and light signaling [20, 35, 39] through its interaction with other bHLH transcription factors that negatively regulate cell elongation, as AtIBH1, AIFs and HFR1 [35, 39, 64]. In *Arabidopsis thaliana*, a triantagonistic bHLH system cascade negatively regulates cell elongation in response to multiple hormonal and environmental signaling pathways [43]. In this system, the homodimer HBI1:HBI1 is directly bound to the promoter of two EXPANSIN genes activating its transcription. Otherwise, the interaction of IBH1 with HBI1 inhibits, by heterodimerization, the production of the activator homodimer which in turn determines the repression of both EXPANSIN genes. In addition, PRE1 activates the DNA binding capacity of HBI1 by sequestering its inhibitor IBH1 throughout the PRE1:IBH1 heterodimer formation [43]. Also, this triantagonistic system has been demonstrated in the interactions between the bHLH Activator of Cell Elongation 1(ACE1) and two atypical HLH proteins, AtIBH1 and PRE1, in *Arabidopsis* [31] and between ACE1 and ATBS1 interaction factors (AIF2, AIF3 and AIF4) or PRE1 in response to BRs and light [32]*.* Likewise, through a similar regulatory system, the ARF/BZR/PIF interaction stimulates the hypocotyl elongation in *Arabidopsis* [38].

Apparently, in strawberry, *FaPRE1* does not play a similar function to that of *SlPRE2* and cannot be considered an orthologous gene. However, its function is clearly involved in the fruit ripening process. In fact, the genes regulated by *FaPRE1* are different to those regulated by *SlPRE2*.

## Conclusions

In summary, this work presents, for the first time in strawberry ripened fruits, experimental data that support an important novel function for the atypical HLH *FaPRE1* during fruit ripening. FaPRE1 antagonistically coordinated the transcription of genes related to both receptacle growth and ripening. Thus FaPRE1, in ripened receptacle, represses the transcription of receptacle growth promoting genes while activating the transcription of those genes related to the receptacle ripening process.

## Methods

### Plant material

*Fragaria × ananassa* Duch. (cv. Camarosa) plants were grown under field conditions in Huelva (S.W. Spain). Strawberry fruits and achenes were harvested at different stages of development and ripening: small-sized green fruits (G1, 2–3 g), middle-sized green fruits (G2, 3–5 g), full-sized green fruits (G3, 4–7 g), white fruits (W, 5–8 g), full-ripe red fruits (R, 10–20 g), over-ripe fruits (OR, 10–20 g) and senescent fruits (SN, 10–20 g). Flowers, floral buds, petals and vegetative tissues such as pedicels and expanding leaves were also collected. *Nicotiana benthamiana* and strawberry plants (*F. × ananassa* Duch. cv. Elsanta) used for infiltration were grown in plant chamber at 25 °C, 10.000 lx and 80% humidity. All tissues analyzed were immediately frozen in liquid nitrogen and then stored at − 80 °C. The strawberry plants were acquired in “Viveros California S.L.” (Huelva, Spain) while *Nicotiana benthamiana* seeds were a gift from Dr. Muñoz-Alamillo.

### Hormonal treatments

With the objective to eliminate the auxins of the fruit, the achenes were carefully removed from two sets of 50 full-sized green fruits (G3) each, in accordance with [9]. Briefly, one set of de-achened G3 fruits was covered with lanolin paste containing indole-3-acetic acid (IAA) 1 mM in 1% (w/v) dimethyl sulphoxide (DMSO). The other group of de-achened fruits (control group) was covered with the same paste but without IAA. Sample collection and analysis were performed following the instructions by [9].

For the gibberellic acid (GA_3_) treatment, strawberry fruits were injected at G2 stage with paclobutrazol (PBZ) 100 μM and GA_3_100 μM. PBZ is a triazole that slows plant growth blocking the synthesis of gibberellins [65]. Control fruits were injected with water. For each treatment, 30 fruits were infiltrated. Fruits were harvested after 24 h of experimentation. In parallel, in vitro strawberry plants (*F. × ananassa* Duch. cv. Chandler), micropropagated in a N30K medium supplemented with 2.2 mM kinetin [66], were treated with gibberellic acid. Two groups of six independent clones were grown in MS medium supplemented with GA_3_ (100 μM) and PBZ (100 μM), respectively, and kept in a growth chamber for 11 days prior harvest. Untreated plants were used as control. All samples and tissues after collection were immediately frozen in liquid nitrogen and then stored at − 80 °C.

In order to block ABA biosynthesis, 20 strawberry fruits (*F.* x *ananassa* cv. Elsanta) in G3 stage of development were injected with nordihydroguaiaretic acid (NDGA) 100 μM. NDGA is an ideal inhibitor of the 9-*cis*-epoxycarotenoid dioxygenase (NCED) enzyme activity [67] and it has previously been demonstrated to decrease endogenous ABA concentration in ripe fruit receptacle [9]. The fruits were injected with 1–2 ml of NDGA solution or water (control fruits) and harvested after 8d of treatment, frozen in liquid nitrogen and stored at − 80 °C until use. These samples were used for measurement of the ABA content and relative expression of *FaPREs* and *FaNCED1* genes.

### Quantification of abscisic acid content

Deuterated abscisic acid (dABA) was used as an internal standard. Both the dABA preparation and ABA extraction from strawberry samples were performed following the instructions by [9]. In order to determine the ABA amount, a HPLC-MS system (VARIAN 1200 L Triple Quadrupole) was used with a column (150 × 2.1 mm i.d. Phenomenex C_18_ with 3 μm particle) (California, USA). The conditions and procedure used for the analysis were the same described by [9].

### Bioinformatic resources

Resources of National Center for Biotechnology Information (NCBI) (Bethesda, MD) (http://www.ncbi.nlm.nih.gov) and the European Bioinformatics Institute server (EBI) (http://www.ebi.ac.uk/) were used for in silico study of *FaPRE* genes sequences against databases. Multiple sequence alignment and phylogenetic tree construction were performed with the EBI ClustalW2 program or the MegAlign program (from the Lasergene DNASTAR software package) as well as the FigTree program (http://tree.bio.ed.ac.uk/software/figtree/). The prediction of domains and functional sites was performed with an InterProScan database (version 4.8) (www.ebi.ac.uk/Tools/pfa/iprscan/) and the prediction of protein localization sites in cells was performed with a Plant-mPLoc computer program (http://www.csbio.sjtu.edu.cn/cgi-bin/PlantmPLoc.cgi). BlastN was also used to localize the genes position in *F. vesca* and *F.* x *ananassa* genome at a GDR databank (https://rosaceae.org). Available *F. vesca* (v 2.0.a2) genome [44] were used to determine *FaPRE* promoter sequences. The promoter analysis of *FaPRE* genes were performed with the PlantCARE database (http://bioinformatics.psb.ugent.be/webtools/plantcare/html/).

### Generation of RNAi constructs and transfection of strawberry fruits by agroinfiltration

A fragment of 626-pb (RNAi-fragment) from *FaPRE1* cDNA was PCR amplified and cloned into pCR®8/GW/TOPO® vector (Invitrogen). Later, the RNAi-fragment was transferred to the pFRN binary vector (courtesy of Marten Denekamp) by LR recombination. The RNAi construct (pFRN-*FaPRE1*) generated was tested by sequencing and restriction analyses prior to transformation of strawberry fruit. The RNAi-*FaPRE1* construct was transformed into *Agrobacterium tumefaciens* strain AGL1. RNAi-construct was used to obtain transient transgenic strawberry fruit with the *FaPRE1* expression silenced by agroinfiltration [68]. The injection of RNAi-construct was performed with a syringe into the base on the entire fruits attached to the strawberry plant following the indications of [68]. 30–40 fruits were inoculated and analysed of a total of 15–25 strawberry plants.

### Subcellular localization analysis

The construct used for localization studies was derived from the binary vector pK7WGF2, which allows for the N-terminal fusion of the selected protein with GFP [69]. The 282-bp CDS of the strawberry *FaPRE1* gene was amplified from *F.* x *ananassa* cDNA using specific primers (Additional file 12) and cloned into the pDONR™221. The PCR-product was then transferred to the pK7WGF2 destination vector, resulting in *35S*-*GFP*-*FaPRE1* fusion construct. The generated construct was tested through sequencing prior to *N. benthamiana* leaves agroinfiltration. The procedures used for *N. benthamiana* agroinfiltration have been previously described [8, 10]. *N. benthamiana* plants were agroinfiltrated with clones to express FaPRE1-GFP and GFP. The samples were imaged 2 days after agroinfiltration on a Leica TCS SP8 point scanning confocal microscope using the pre-set settings for GFP with Ex:488 nm, Em:500-550 nm. For nuclear staining, samples were stained with a solution of 40 μg/ml DAPI 10 min before imaging with Ex:405 nm, Em: 448–525 nm.

### RNA isolation

Total RNA was isolated from three independent pools (10 fruits per pool) of strawberry fruits at different development stages and plant vegetative tissues following the indications of [70]. When strawberry fruits were used, the achenes were always removed before extracting the RNA from the samples. In any case, the RNA extracted was always incubated with DNase I (RNase free) (Invitrogen) to eliminate the genomic DNA contamination following manufacturer’s instructions. The RNA quality and integrity were checked using an Agilent 2100 Bioanalyzer (Agilent Technologies, Deutschland). Only samples with a RIN value ≥8 were used for subsequent transcriptomic analyses.

### Microarray generation and analysis

The transcriptomic changes produced by the *FaPRE1* silencing were determined using a custom-made oligo microarray platform (60-mer length; FraGenomics 35 k) containing a total of 34.616 singletons corresponding to those sequences published in the strawberry genome project (http://www.strawberry.org). We compared the transcriptomes from control red receptacles injected with the empty pFRN vector versus red receptacles injected with the RNAi*-FaPRE1* construct. The same microarray platform was also used for the transcriptomic analysis of the strawberry ripening fruit process comparing the transcriptomes from green (G1) receptacle versus red (R) receptacle [19]. The corresponding data were deposited in the GEO database (www.ncbi.nlm.nih.gov/geo/) with the GSE125995 for silencing data and GSE126220 for ripening data [19]. The criteria for the selection of the differentially expressed genes were log_2_ fold change > ±2 and *p* ≤ 0.05 in both analyses. The microarray characteristics, hybridization and processing conditions were as described in [8].

### Validation of microarray data and expression analysis by quantitative real-time PCR

Expression analyses of the genes herein studied in different physiological conditions and for microarray validation were performed by quantitative real-time PCR (qRT-PCR) using iCycler system (BioRad), as previously described by [9, 71]. Specific primers of the 3’UTR regions were designed to analyze the expression of the *PRE-*like genes (*FaPRE1*, *FaPRE2* and *FaPRE3*) identified in the strawberry genome. Besides, to validate the expression data obtained in the microarray analysis, specific primers were designed on several genes that showed differential expression in the experimental situations analyzed. Additional file 12 depicts the primer sequences used for all quantitative amplifications. The relative increase or decrease of gene expression in the samples in comparison to that in the control gene was calculated in accordance with Pedersen and [72]. Interspacer *26S–18S* gene was selected as control gene owing to its constitutive expression.

### Statistical analysis of data

Statistical significance was tested with a Student’s *t*-test using SPSS software.

## Supplementary information

**Additional file 1.** Phylogenetic tree of 184 bHLH/HLH transcription factors. FaPREs taxa is written in black and grey clade contains sequences belonging to subgroup 16. The tree was constructed using the IQTREE web software (http://iqtree.cibiv.univie.ac.at/) by the neighbor-joining method with 1000 bootstrap replicates.

**Additional file 2.** Subfamily classification of 182 plant bHLH/HLH sequences examined in this study and additional information.

**Additional file 3.** A. Table containing additional information of the atypical HLH sequences belonging to subgroup 16. B. Screenshot corresponding to the prediction of domains performed with InterProScan database (version 5) (http://www.ebi.ac.uk/Tools/pfa/iprscan5). C. Sequence alignment of bHLH proteins. Identical amino acids are shaded in black. The two helices are indicated with sets of black arrows and the loop is indicated with a grey line. Numbers indicate amino acid positions. D. Screenshot corresponding to the result of protein localization sites prediction in cells performed with the Plant-mPLoc computer program (http://www.csbio.sjtu.edu.cn/cgi-bin/PlantmPLoc.cgi).

**Additional file 4. **Phenotypic analysis of *F. × ananassa* “Chandler” in vitro plants grown in N30K medium supplemented with hormones. (A) General view of control plants (CONTROL) and treated plants with gibberellic acid (GA_3_) and paclobutrazol (PBZ) after 11 dpt. Analysis by qRT-PCR of *FaPRE1*, *FaPRE2* and *FaPRE3* expression in leaves (B), pedicels (C) and roots (D) from in vitro strawberry plants (*F. × ananassa* “Chandler”) treated with GA_3_ and PBZ. Mean values ± SD of three independent experiments are shown. CONTROL, plants in N30K medium; GA_3_, plants in N30K medium supplemented with GA_3_ 100 μM; PBZ, plants in N30K medium supplemented with paclobutrazol 100 μM. Statistical significance with respect to the reference sample (Control) was determined by the Student’s *t*-test: *p < 0.05.

**Additional file 5. **Analysis by qRT-PCR of *FaPRE1*, *FaPRE2* and *FaPRE3* gene expression in strawberry transgenic receptacle agroinfiltrated with the RNAi-*FaPRE1* construct. Control: receptacle agroinfiltrated with the empty pFRN vector; Pool 1, 2 and 3: receptacles agroinfiltrated with *FaPRE1*-pFRN construct.

**Additional file 6 **Total microarray data from transcriptomic comparison between transgenic receptacles agroinfiltrated with *FaPRE1*-RNAi construct and no-transgenic control receptacles. Gene ID and corresponding annotations as reported in *Fragaria vesca* Genome Database (https://www.rosaceae.org/) [73].

**Additional file 7 **Expression data of selected genes in *FaPRE1*-silenced receptacles obtained by QRT-PCR and microarray analysis.

**Additional file 8 **Venn diagrams showing the number of genes down-regulated (A) and up-regulated (B) in strawberry *FaPRE1*-RNAi receptacles respectively and up-regulated in strawberry red receptacle.

**Additional file 9 **All the genes down-regulated in strawberry *FaPRE1*-RNAi receptacles (fold change RNAi) and up-regulated in strawberry red receptacle (fold ripening). Gene ID and corresponding annotations as reported in *Fragaria vesca* Genome Database (https://www.rosaceae.org/) [73].

**Additional file 10 **All the genes up-regulated in strawberry *FaPRE1*-RNAi receptacles (fold change RNAi) and down-regulated in strawberry red receptacle (fold ripening). Gene ID and corresponding annotations as reported in *Fragaria vesca* Genome Database (https://www.rosaceae.org/) [73].

**Additional file 11 **Phylogenetic tree of some functionally characterized atypical HLH transcription factors. The length of each pair of branches represents the distance between sequence pairs, while the units at the bottom of the tree indicate the number of substitution events. FaPRE1(KM655802; *Fragaria x ananassa*); FaPRE2 (XM_004296502; *F. x ananassa*); FaPRE3 (XM_004297270; *F. x ananassa*); SlStyle2.1 (NM_001247361; *Solanum lycopersicum*) [42]; SlPRE2 (XP_004233358.1; *S. lycopersicum*) [34]; AtPRE1 (At5g39860; *Arabidopsis thaliana*) [20]; BNQ3 (NP_190355.2; *A. thaliana*) [62]; KIDARI (NP_849712; *A. thaliana*) [35]; ATBS1 (NP_177590; *A. thaliana*) [32]; AtPRE3 (At1g74500; *A. thaliana*) [30]; PGL2 (Os02g0747900; *Oryza sativa*) [40]; AIF2 (At3g06590; *A. thaliana*) [36]; AIF3 (At3g17100; *A. thaliana*) [36]; AIF4 (At1g09250; *A. thaliana*) [36]; PAR1 (At2g42870; *A. thaliana*) [74]; PAR2 (At3g58850; *A. thaliana*) [74]. Sequences were aligned using MegAlign (MegAlign 5.00; DNASTAR).

**Additional file 12.** Primer sequences used in this work. Fw: forward; Rv: reverse. Up: upper; Low: lower.

## Data Availability

The datasets generated and analyzed during the current study are available in the GEO repository (www.ncbi.nlm.nih.gov/geo/) (GSE125995 for silencing data and GSE126220 for ripening data). The data are public from October 18, 2019.

## Abbreviations

2,4-D: 2,4-dichlorophenoxyacetic acid
ABA: Abscisic acid
bHLH: Basic helix-loop-helix
CDS: Coding DNA sequence
dABA: Deuterated abscisic acid
DAPI: 4′6-diamino-2-phenylindole dihydrochloride
G1: Green1 stage
G2: Green2 stage
G3: Green3 stage
GA_3_: Gibberellic acid
GFP: Green fluorescent protein
HLH: Helix-loop-helix
NCED: 9-*cis*-epoxycarotenoid dioxygenase
NDGA: Nordihydroguaiaretic acid
OR: Overripe stage
PBZ: Paclobutrazol
PRE1: Paclobutrazol resistance 1
qRT-PCR: Quantitative real time PCR
R: Ripe stage
RNAi: RNA interference
SN: Senescent stage (the seven subjective stages of strawberry fruit development)
W: White stage
