# Supplementary figures and images for: An atypical HLH transcriptional regulator plays a novel and important role in strawberry ripened receptacle
Source: BMC Plant Biol. 2019 Dec 27;19:586. doi: 10.1186/s12870-019-2092-4 (PMC6933692; doi:10.1186/s12870-019-2092-4)

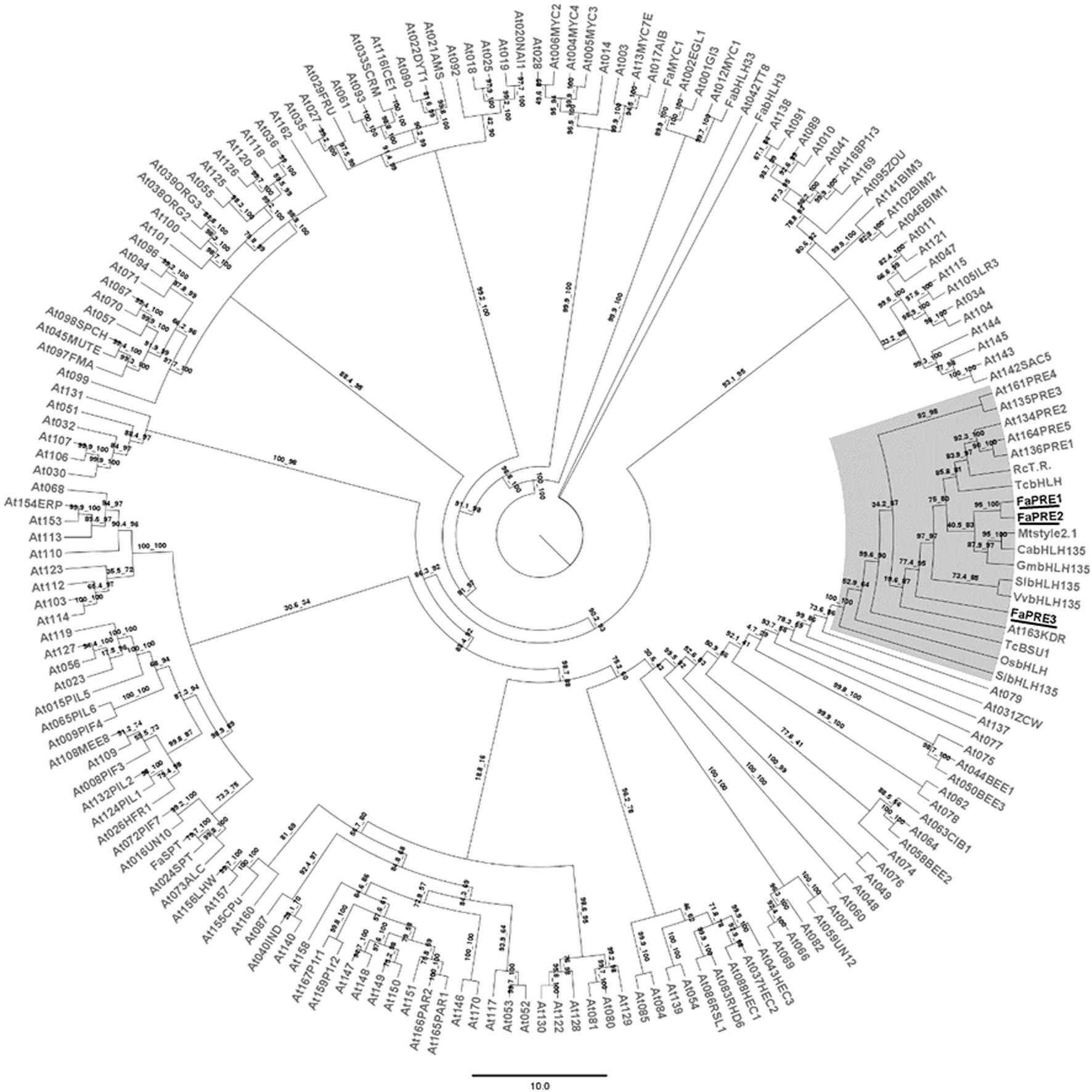

Supplement: Supplementary file 1 — Additional file 1. Phylogenetic tree of 184 bHLH/HLH transcription factors. FaPREs taxa is written in black and grey clade contains sequences belonging to subgroup 16. The tree was constructed using the IQTREE web software (http://iqtree.cibiv.univie.ac.at/) by the neighbor-joining method with 1000 bootstrap replicates. [file 12870_2019_2092_MOESM1_ESM.tif]

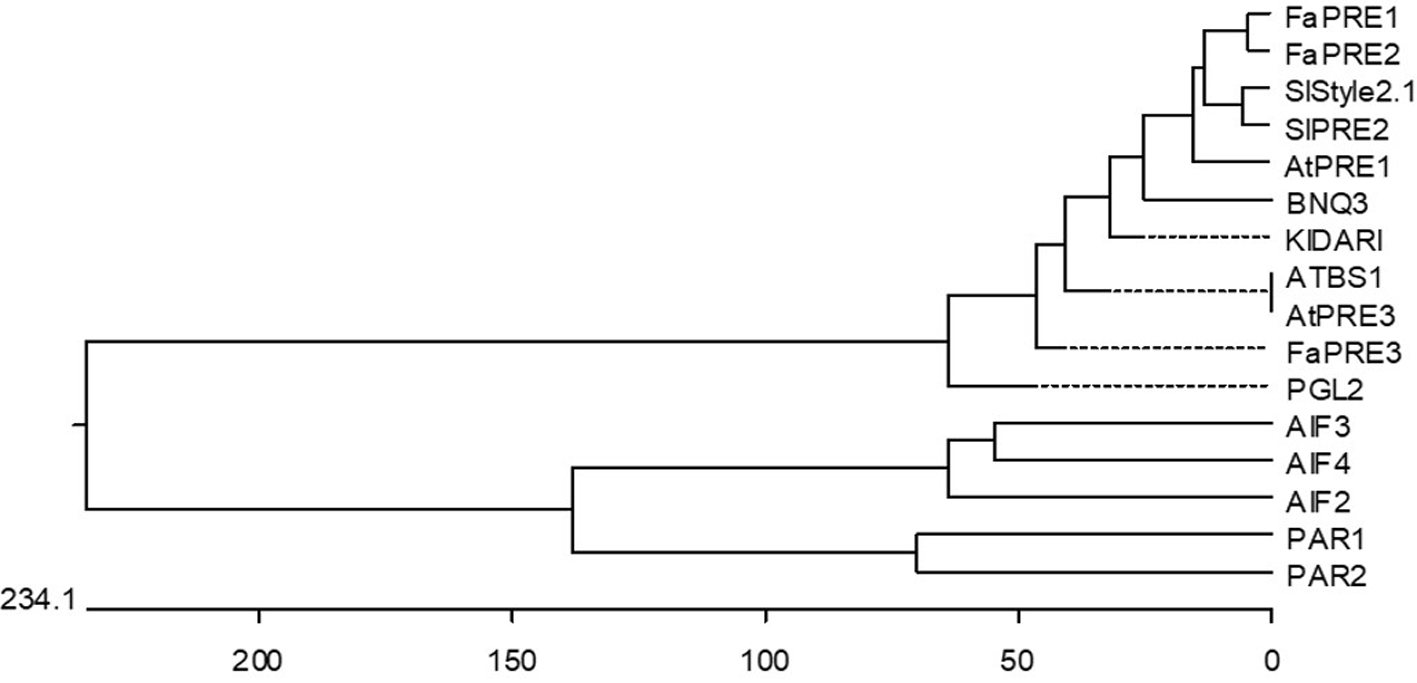

Supplement: Supplementary file 3 — Additional file 3. A. Table containing additional information of the atypical HLH sequences belonging to subgroup 16. B. Screenshot corresponding to the prediction of domains performed with InterProScan database (version 5) (http://www.ebi.ac.uk/Tools/pfa/iprscan5). C. Sequence alignment of bHLH proteins. Identical amino acids are shaded in black. The two helices are indicated with sets of black arrows and the loop is indicated with a grey line. Numbers indicate amino acid positions. D. Screenshot corresponding to the result of protein localization sites prediction in cells performed with the Plant-mPLoc computer program (http://www.csbio.sjtu.edu.cn/cgi-bin/PlantmPLoc.cgi). [file 12870_2019_2092_MOESM3_ESM.tif]

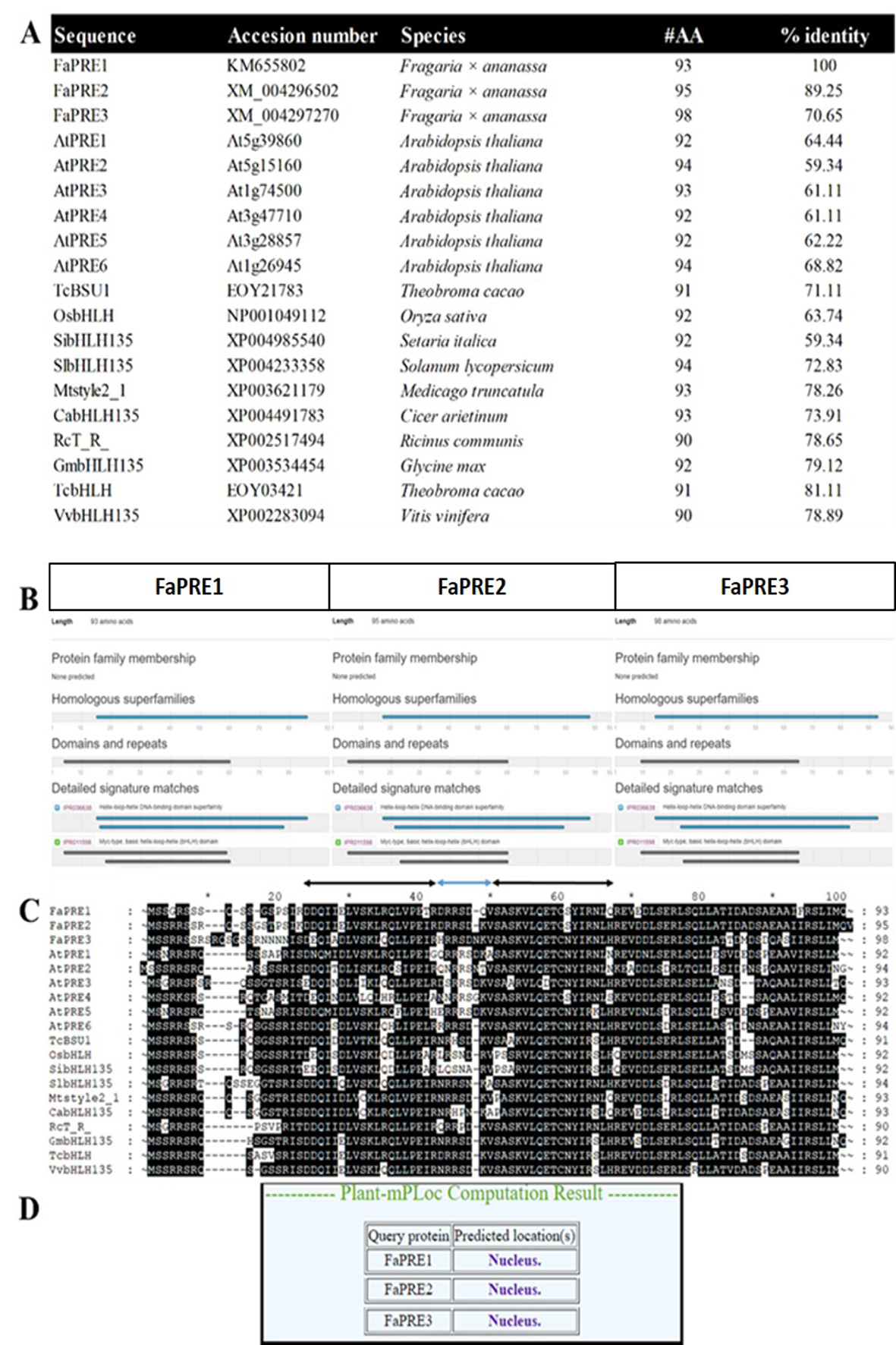

Supplement: Supplementary file 6 — Additional file 6 Total microarray data from transcriptomic comparison between transgenic receptacles agroinfiltrated with FaPRE1-RNAi construct and no-transgenic control receptacles. Gene ID and corresponding annotations as reported in Fragaria vesca Genome Database (https://www.rosaceae.org/) [73]. [file 12870_2019_2092_MOESM6_ESM.tif]

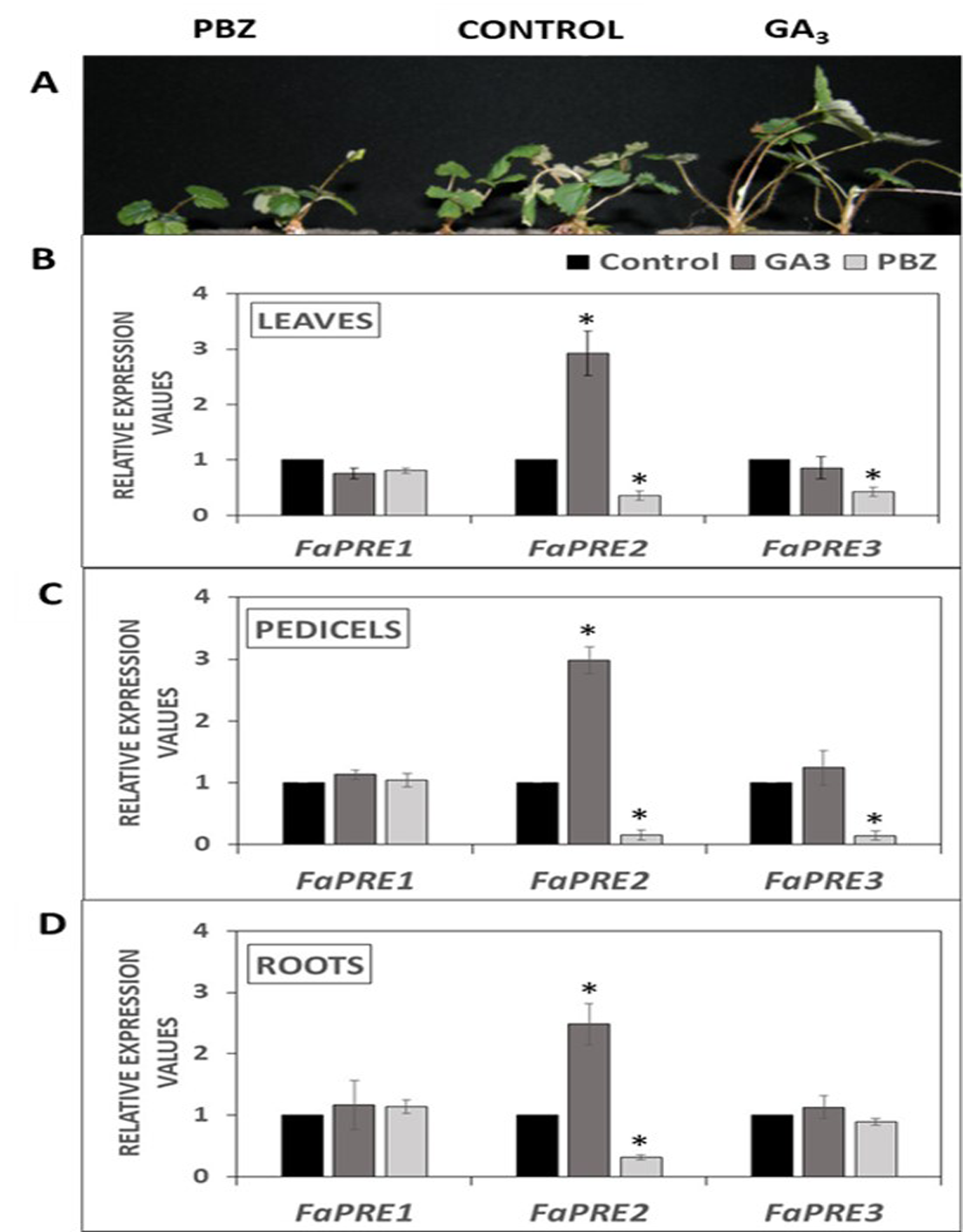

Supplement: Supplementary file 7 — Additional file 7 Expression data of selected genes in FaPRE1-silenced receptacles obtained by QRT-PCR and microarray analysis. [file 12870_2019_2092_MOESM7_ESM.tif]

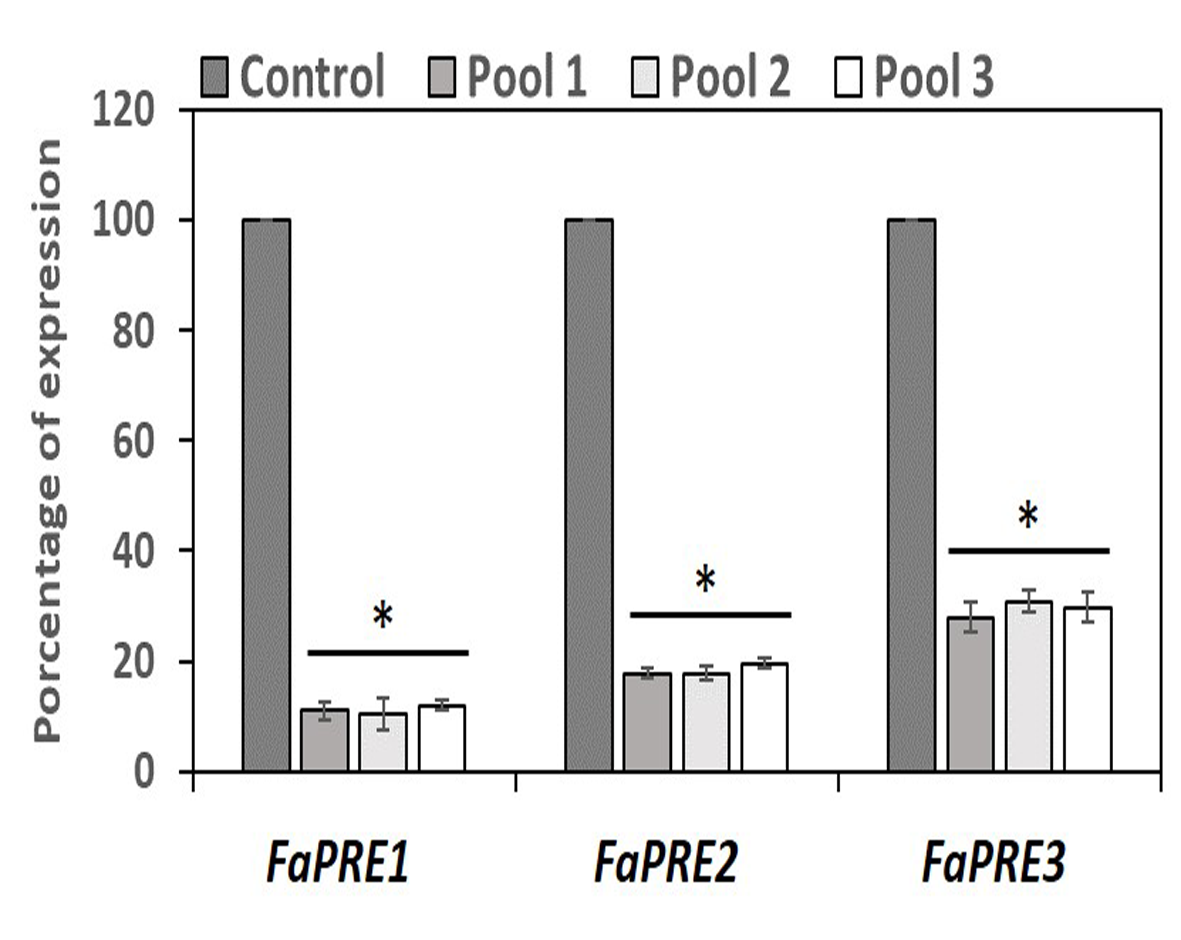

Supplement: Supplementary file 8 — Additional file 8 Venn diagrams showing the number of genes down-regulated (A) and up-regulated (B) in strawberry FaPRE1-RNAi receptacles respectively and up-regulated in strawberry red receptacle. [file 12870_2019_2092_MOESM8_ESM.tif]

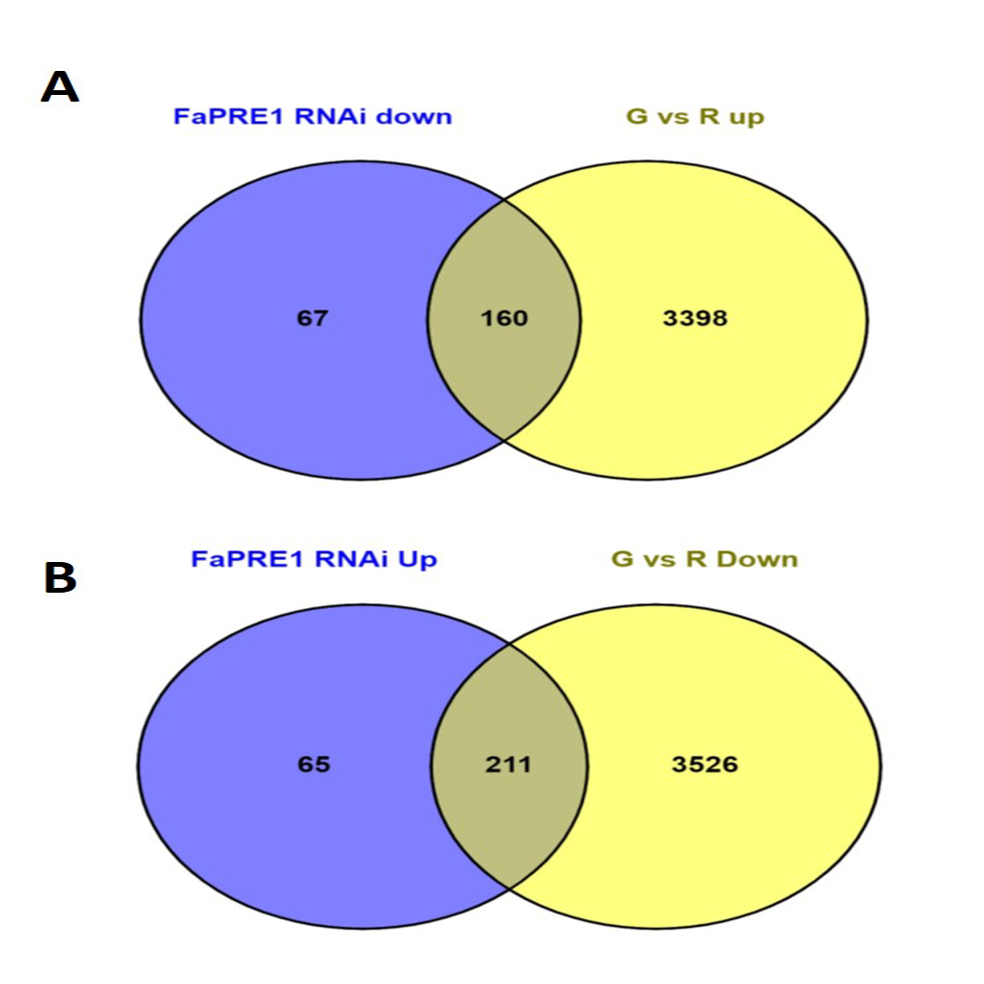

Supplement: Supplementary file 12 — Additional file 12. Primer sequences used in this work. Fw: forward; Rv: reverse. Up: upper; Low: lower. [file 12870_2019_2092_MOESM12_ESM.tif]
